# YpT1-2N0 rectal cancer after neoadjuvant chemoradiation has lower survival compared with pT1-2N0 rectal cancer

**DOI:** 10.18632/oncotarget.5379

**Published:** 2015-10-21

**Authors:** Jue-feng Wan, Ji Zhu, Gui-chao Li, Wen-jie Sun, Zhen Zhang

**Affiliations:** ^1^ Department of Radiation Oncology, Fudan University Shanghai Cancer Center, Fudan University, Shanghai, China; ^2^ Department of Oncology, Shanghai Medical College, Fudan University, Shanghai, China

**Keywords:** rectal cancer, adjuvant chemotherapy, risk factors, good response, SEER

## Abstract

Pathologic T1-2N0 rectal cancer shows an excellent prognosis without preoperative or postoperative chemoradiation. However, oncologic outcome of ypT1-2N0 remains unclear and undetermined. Thus, the aim of this study was to compare the survival of ypT1-2 and pT1-2 rectal cancer patients after radical resection and identify risk factors of ypT1-2 rectal cancer in Surveillance, Epidemiology, and End Results Program (SEER)-registered rectal cancer patients. The results showed that ypT1-2N0 rectal cancer after neoadjuvant chemoradiation has lower survival compared with pT1-2N0 rectal cancer and mucinous/signet-ring cancer and less than 12 lymph nodes retrieval were two risk factors in ypT1-2 patients. These results suggest that ypT1-2 patients with one or two risk factors may benefit from postoperative adjuvant chemotherapy.

## INTRODUCTION

Colorectal cancer is one of the most common cancers in the western world and preoperative chemoradiotherapy (CRT) followed by total mesorectal excision is the standard of care for patients with locally advanced rectal cancer [1–5]. Park et al. revealed that treatment response to neoadjuvant chemoradiotherapy was an early surrogate marker and tumor response was associated with 5-year recurrence free survival [6]. Although it is well known that complete pathologic response to chemoradiation is associated with excellent prognosis, there are few studies evaluating the oncologic outcome of patients with ypT1-2N0 rectal cancer who underwent preoperative chemoradiation.

Actually, pathologic T1-2N0 rectal cancer shows an excellent prognosis without preoperative or postoperative chemoradiation. However, oncologic outcome of ypT1-2N0 remains unclear and undetermined. Thus, the aim of this study was to compare the cancer specific survival of ypT1-2 and pT1-2 rectal cancer patients after radical resection and identify risk factors of ypT1-2 rectal cancer.

## RESULTS

### Patient characteristics

We identified 10,673 eligible elderly patients in SEER database during the 9-year study period (between 2004 and 2012), which included 8,433 patients in pT1-2 and 2,240 patients in ypT1-2. There were 6167 (57.8%) males and 4506(42.2%) females. Patient demographics and pathological features are summarized in Table 1.

**Table 1:** Patient characteristics

Variable	Total	pT1-2	ypT1-2	*P* value
	*n* = 10,673	*n* = 8,433	*n* = 2,240	
Sex				<0.001
Male	6167	55.9%	64.9%	
Female	4506	44.1%	35.1%	
Race				<0.001
White	8836	83.1%	81.6%	
Black	796	6.9%	9.5%	
Other	1041	10%	8.9%	
Pathological grading				0.048
High/Moderate	8603	81%	79%	
Poor/undifferentiation	804	7.3%	8.6%	
Unknown	1266	11.7%	12.4%	
Histological type				<0.001
Adenocarcinoma	10465	98.4%	97%	
Mucinous/Signet ring cell	208	1.6%	3%	
Stage				<0.001
pT1+ypT1	6509	66.9%	38.3%	
pT2+ypT2	4164	33.1%	61.2%	
No. of LNs dissected				<0.001
<12	7013	66.6%	62.2%	
≥12	3660	33.4%	37.8%	

### Clinicopathological differences between the two groups

When compared among two subgroups, it was investigated that significant differences were found among the sex (more female in pT1-2, *p* < 0.001), race, pathological grading, histological type (more mucinous/signet ring cell in ypT1-2, *p* < 0.001), stage (more pT1 in pT1-2, *p* < 0.001) and current standard (more cases with ≥ 12 LNs dissected in ypT1-2, *p* < 0.001). (Table 1).

### Cancer specific survival between the two groups

**Figure 1:**
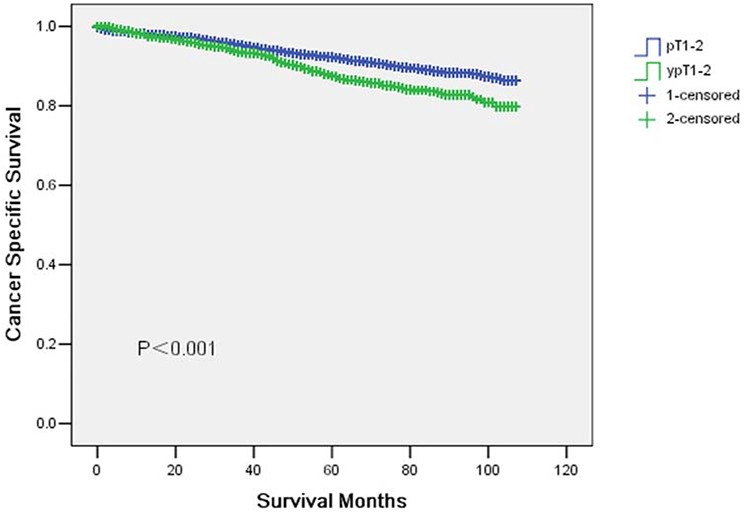
Cancer specific survival curves in yT1-2 and ypT1-2 rectal cancer patients The 5-year cancer specific survival (CSS) was 92.2% in pT1-2 and 87.5% in ypT1-2 (*p* < 0.001).

**Figure 2:**
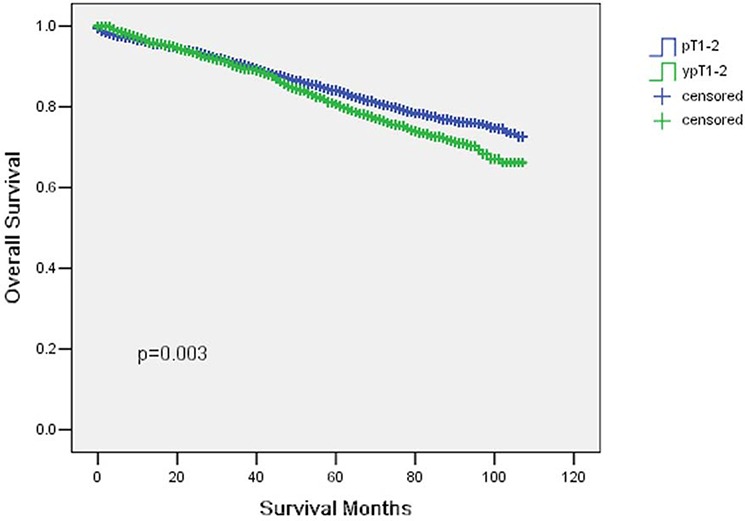
Overall survival curves in yT1-2 and ypT1-2 rectal cancer patients The 5-year overall survival (OS) was 84% in pT1–2 and 80.6% in ypT1-2 (*p* = 0.003).

The 5-year CSS was 92.2% in pT1-2 and 87.5% in ypT1-2 and the 5-year overall survival was 84% in pT1-2 and 80.6% in ypT1-2, which had significant difference in univariate log-rank test (*P* < 0.001 and *P* = 0.003, respectively) (Fig. 1 and 2). Besides, black race (*P* < 0.001), poor or undifferentiation tumor grade (*P* = 0.002), mucinous/signet-ring cancer (*P* < 0.001), pT2(*P* < 0.001), and less number in LNs dissection (*p* = 0.001) were identified as significant risk factors for poor survival on univariate analysis (Table 2). When multivariate analysis with Cox regression was performed, we convinced the above five factors also as independent prognostic factors (Table 2).

**Table 2:** Univariate and multivariate survival analyses of rectal cancer patients according to various clinicopathological variables

Variable	*n*	5-year CSS (%)	Univariate P	Multivariate P
Sex			0.899	0.938
Male	6167	91		
Female	4506	91.4		
Race			<0.001	<0.001
White	8836	91.1		
Black	796	87.5		
Other	1041	94		
Pathological grading			0.002	0.015
High/Moderate	8603	91.4		
Poor/undifferentiation	804	86.9		
Unknown	1266	92.3		
Histological type			<0.001	0.007
Adenocarcinoma	10465	91.4		
Mucinous/Signet ring cell	208	81.8		
Stage			<0.001	<0.001
pT1+ypT1	6509	93.3		
pT2+ypT2	4164	87.9		
No. of LNs dissected			0.001	<0.001
<12	7013	90.6		
≥12	3660	92.3		
Stage			<0.001	0.004
pT1–2	8433	92.2%		
ypT1-2	2240	87.5%		

### Potential risk factors and prognostic significance in ypT1-2

**Figure 3:**
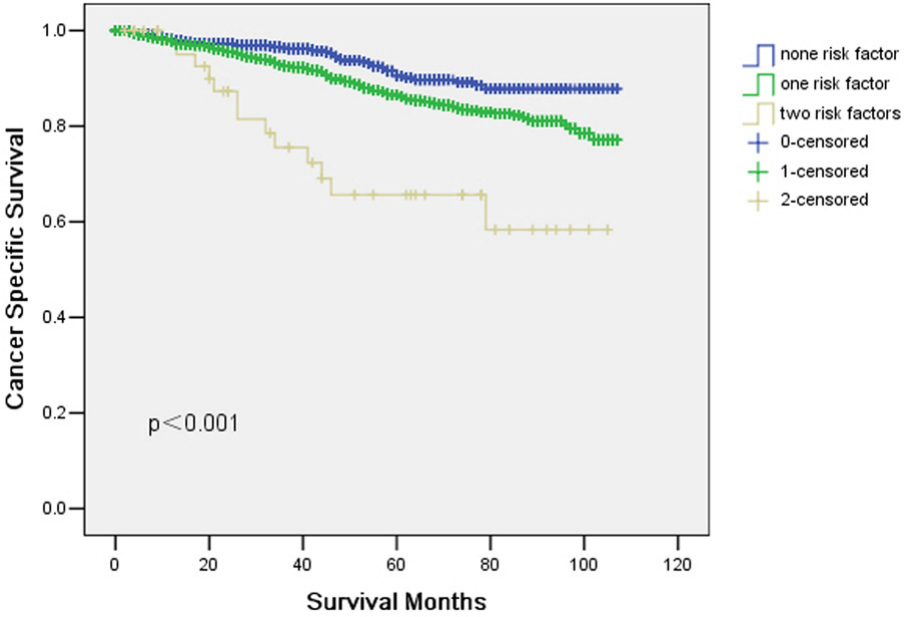
Cancer specific survival (CSS) in ypT1-2 rectal cancer patients according to number of risk factors The 5-year cancer specific survival (CSS) in patients with none, one or two risk factors was 90.5%, 86.5% and 65.6%, respectively (*p* < 0.001).

All potential risk factors, including gender, race, pathological grading, histological type, stage and No. of LNs dissected were evaluated by using the Kaplan-Meier method (compared with Log rank test). Among these potential risk factors, race, histological type and No. of LNs dissected exhibited a correlation with CSS (Table 3). Cox multivariate regression analysis revealed only two factors to be associated with CSS: histological type and No. of LNs dissected (Table 3). The 5-year CSS in patients with none, one or two risk factors was 90.5%, 86.5% and 65.6%, respectively (*p* < 0.001) (Fig. 3).

**Table 3:** Univariate and multivariate survival analyses of ypT1-2 rectal cancer patients according to various clinicopathological variables

Variable	*n*	5-year CSS (%)	Univariate P	Multivariate P
Sex			0.75	0.615
Male	1453	86.6		
Female	787	89.2		
Race			0.009	0.352
White	1828	87.6		
Black	211	81		
Other	201	93.6		
Pathological grading			0.052	0.861
High/Moderate	1769	88.3		
Poor/undifferentiation	193	79.9		
Unknown	278	88		
Histological type			<0.001	<0.001
Adenocarcinoma	2171	88		
Mucinous/Signet ring cell	69	71.2		
Stage			0.437	0.647
ypT1	870	87.5		
ypT2	1370	87.4		
No. of LNs dissected			0.001	0.001
<12	1394	85.9		
≥12	846	90.3		

## DISCUSSION

Two retrospective studies investigated the oncologic outcomes in patients with ypT1-2N0 rectal cancer who underwent CRT and radical surgery and compare with those who did not receive preoperative CRT [10–11]. They found similar results that no significant difference was observed in the 5-year local recurrence rate and overall survival for two groups. However, the number of patients in these two studies is too small to provide adequate power for drawing any definitive conclusions regarding oncologic outcomes.

In the present study, we identified 8,433 patients in pT1-2 and 2,240 patients in ypT1-2. The 5-year CSS was 92.2% in pT1-2 and 87.5% in ypT1-2 (*P* < 0.001). Thus, ypT1-2N0 rectal cancer after neoadjuvant chemoradiation has lower survival compared with pT1-2N0 rectal cancer. The standard treatment for T1-2N0 disease is surgery alone without preoperative or postoperative CRT. Actually, pathologic T1-2N0 rectal cancer shows an excellent prognosis without postoperative chemoradiation. In contrast, clinical practice guideline of adjuvant chemotherapy of ypT1-2 rectal cancer is not based on solid evidence and the level of scientific evidence for sufficient benefit is much lower than colon cancer [12–15].

The recently reported meta-analysis of 21 RCTs showed that a significant reduction in the risk of death (17%) and in the risk of disease recurrence (25%) among patients with rectal cancer undergoing adjuvant chemotherapy as compared to those undergoing observation [16]. However, only one of these 21 RCTs contained patients treated with preoperative chemoradiotherapy and almost all of these patients underwent curative resection of rectal cancer without preoperative treatment. However, things are more complicated in the era of the wide use of neoadjuvant chemoradiation for patients with locally advanced rectal cancer.

The European Organization for Research and Treatment of Cancer (EORTC) 22921 trial did not confirm a significant disease-free or overall survival benefit for adjuvant FU-based chemotherapy for locally advanced rectal cancer [17]. A second analysis of the EORTC 22921 trial was performed to find whether there is a subset of patients who, after preoperative radiotherapy or chemoradiotherapy and surgery, may benefit from adjuvant postoperative FU/leucovorin chemotherapy. Exploratory analyses suggest that only good-prognosis patients (ypT0–2) benefit from adjuvant chemotherapy [18].

However, two retrospective studies did not find patients with ypT1-2 benefit from postoperative adjuvant chemotherapy [19–20]. Fietkau et al. showed that 3-year disease free survival (DFS) for patients without lymph node metastases (ypN0) was excellent, independent of whether they had received postoperative chemotherapy (87.5 ± 6.0 percent) or not (87.7 ± 6.7 percent). In addition, SEER-Medicare-linked database showed that patients who had already received 5-FU-based neoadjuvant chemoradiotherapy, postoperative 5-FU-based chemotherapy did not prolong cancer-specific survival (CSS) in ypT1-2 (*P* = 0.960).

Thus, Up to date, no general agreement has been reached on the indications of adjuvant chemotherapy for ypT1-2 patients. In our study, we identified histological type and No. of LNs dissected as two significant risk factors for survival on univariate and multivariate analysis. Thus, ypT1-2 patients with one or two risk factors may benefit from postoperative adjuvant chemotherapy.

Although this is a large population-based study, it has several potential limitations. First, the SEER database lacks several important tumor characteristics (eg, perineural invasion, lymphovascular invasion and distance from anal verge). Thus, our analyses could not adjust for these potential confounding factors. Second, our study is the lack of data in the SEER registry on the use of chemotherapy, resulting in a potentially significant confounder in the current study. It is possible that patients may have received adjuvant chemotherapy. Still, our study has its convincing power for its larger population based study.

In conclusion, ypT1-2N0 rectal cancer after neoadjuvant chemoradiation has lower survival compared with pT1-2N0 rectal cancer and ypT1-2 patients with one or two risk factors may benefit from postoperative adjuvant chemotherapy.

## MATERIALS AND METHODS

### Patient selection in the SEER database

The SEER, a population-based reporting system, was surveyed for the retrospective collection of data used in the analysis. The SEER program collects and publishes cancer incidence and survival data from 18 population-based cancer registries, covering >25% of the US population. Because no personal identifying information was used in the analysis, this study was granted an exemption from the Institutional Review Board of the study institution on March 30, 2012.

Cases of rectal cancer (C20.9 Rectum, NOS) from 2004 to 2012 were extracted from the SEER database (SEER*Stat 8.2.1) according to the Site Recode classifications with limitation to radiation prior to surgery and radiation preoperatively and post-surgery. Histological type were limited to adenocarcinoma (ICD-03, 8140/3, 8210/3, 8261/3, 8263/3), mucinous adenocarcinoma (ICD-03, 8480/3), and signet ring cell carcinoma (ICD-03, 8490/3). We selected this range because American Joint Committee on Cancer (AJCC) TMN stage was available since 2004 and chemoradiation has become the standard treatment since the landmark German CAO/ARO/AIO-94 trial using preoperative chemoradiation which was published in 2004. Other exclusion criteria were as follows: more than one primary cancer but the rectal cancer wasn't the first one, synchronous distance metastases, and patients with unknown TNM stage.

### Statistical analysis

Gender, race, pathological grading, histological type, stage, No. of lymph nodes (LNs) dissected and cancer specific survival (CSS) were extracted from SEER database. CSS was calculated from the date of diagnosis to the date of cancer specific death. Deaths attributed to the rectal cancer were treated as events and deaths from other causes were treated as censored observations. The Kaplan-Meier method was used to estimate the CSS [7]. The association between each of the potential prognostic factors and the estimated CSS was tested with the log–rank test [8]. Multivariate analysis was performed using the Cox regression model [9]. The statistical test was two sided and *P* < 0.05 was considered statistically significant. PASW Statistics 13 (SPSS Inc., Chicago, USA) was used for the statistical analysis.
